# The double face of cold in cancer

**DOI:** 10.1016/j.tranon.2022.101606

**Published:** 2022-12-20

**Authors:** Konstantinos Voskarides

**Affiliations:** a Department of Basic and Clinical Sciences, University of Nicosia Medical School, Nicosia, Cyprus; b School of Veterinary Medicine, University of Nicosia, Nicosia, Cyprus

## Abstract

In a recent paper published in *Nature*, multiple evidence is provided that cold exposure causes tumor growth restriction in mice, by activating brown adipose tissue metabolism and by subsequent cancer cells’ glucose starvation. The paper shows a tumor growth inhibition by 80% for multiple cancer types in mice exposed to 4 °C in comparison with mice exposed to 30 °C. These results are very promising since cost effective protocols could be designed for future clinical trials, for several cancer forms. In this commentary, an extensive analysis is performed on the potential of these results. Some previous published studies are discussed as well, showing differences in tumor growth for mice housed in different external temperatures.

## Highlights

• Growth rate of tumors is different at 30 °C, 22 °C and 4 °C.
• Temperature can affect anticancer immunity and tumors’ metabolism.
• Cancer incidence is linearly increased along with low temperatures.

In a recent paper published in *Nature* by Seki et al. [1], authors show that cold causes tumor growth restriction in mice, by activating brown adipose tissue metabolism and by subsequent cancer cells’ glucose starvation. The paper shows a tumor growth inhibition by 80% for multiple cancer types in mice exposed to 4 °C in comparison with mice exposed to 30 °C. These results are very promising since cost effective protocols could be designed for future clinical trials. Here I will discuss some previous published studies, related with tumor growth under different environmental conditions. It is highly significant to consider all the available parameters before proceeding further and take advantage of these results of Seki et al. [1]. In this commentary three lines of previously published evidence will be discussed: (a) Mice that are exposed in mild cold (20–22 °C) have an increased tumor growth and more metastasis events than mice exposed to 30–31 °C, (b) Native human populations living in the coldest regions of our planet (environmental temperatures below −20 °C) have the highest cancer incidence worldwide, (c) Linear regression statistics of 186 countries/populations worldwide show that the lower the environmental temperature (average annual temperature), the higher is the incidence of cancer.

The first line of evidence is coming from Kokolus et al. [2], related with mice experiments. The authors injected tumor cells in two groups of mice, acclimated in 22–23 °C (mild cold) and 30–31 °C, respectively. Tumors were rapidly expanded in the mild-cold mice group. The authors observed a significant reduction in the tumor growth rate in the 30–31 °C group and fewer metastasis events. Results were similar for four different tumor models. They also observed more CD8+ T immune cells in the tumor microenvironment of the 30–31 °C mice group. Additionally, T cells of the 30–31 °C group were metabolically more active expressing more IFN-γ, CD69 and Glut-1. The authors concluded that mild cold may suppress immune response of mice, this increasing tumor growth. The same research group investigated the efficiency of antigen-presenting cells (dendritic cells) in mice exposed to mild-cold [3]. Dendritic cells are crucial for T cell activation and subsequently for cancer cell elimination. They found that activated dendritic cells from tumor bearing mice at mild-cold conditions are less able to stimulate T cell proliferation than the ones from tumor-bearing mice acclimated at ∼30 °C. They concluded that antigen presentation may be suppressed at mild-cold conditions leading to reduced immune surveillance [3]. Seki et al. [1], showed that the anticancer effect that they observed at 4 °C was absent when the mice were exposed to mild-cold at 22 °C. On the other hand, the only cancer patient (Hodgkin's lymphoma) that Seki et al. [1] included in their study, was exposed to mild cold at 22 °C observing reduced tumor glucose uptake, this supporting their hypothesis.

**Fig. 1:**
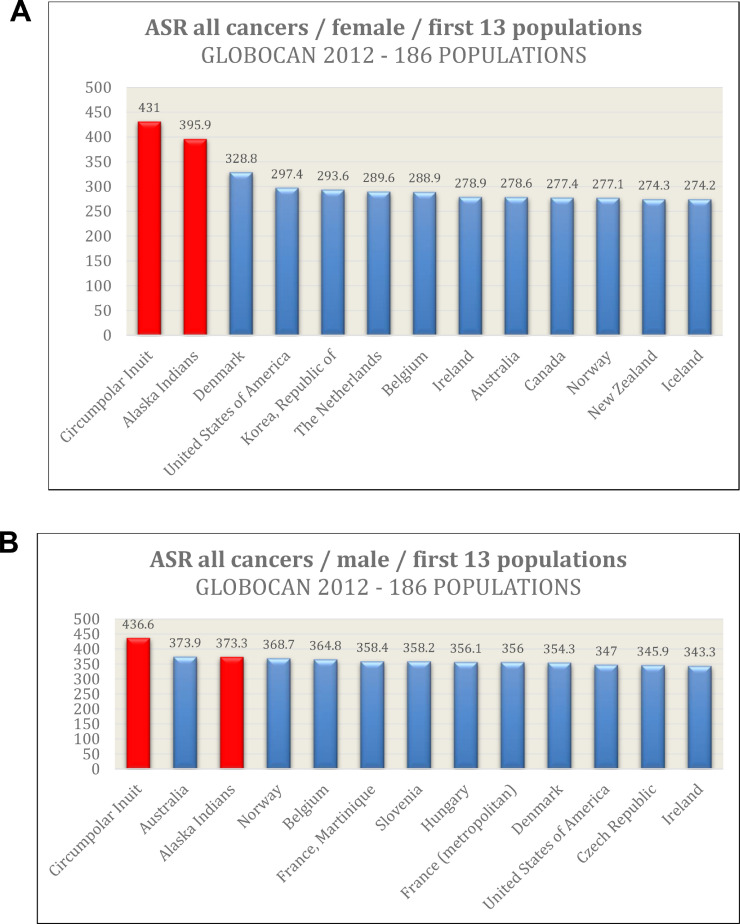
Cancer age-standardized rate (ASR) data of the first 13 ranked populations (A: females, B: males) out of 186 ones. Bars of Inuit and Alaska Indians are in red color. ASR values are according GLOBOCAN-2012 [7] and Young et al. [6].

The second line of evidence is coming from human populations living under extreme cold conditions. Inuit (Canadian and Greenlandic native populations) and Athabascans (Native Americans living mainly in Alaska, known also as Alaska Indians) live at extreme cold environments, often much below −20 °C. These populations exhibit extreme high cancer incidence, probably the highest worldwide (Fig. 1). The incidence is high for all cancer types together and especially for lung, breast, and colorectal cancer [4], [5], [6]. Lung cancer can be attributed to high rates of smoking in these populations. There is also some evidence for high cancer incidence in Siberian Eskimos. Cancer is a major health problem for those arctic populations.

The third line of evidence is related with a geographical pattern, revealing a universal trend for all human populations (published analysis [8] included 186 countries/populations [6,7]). The trend shows that the lower the average annual temperature, the higher is the incidence (age-standardized) of cancer [8]. The relationship is of continual character and the statistical significance is very high under a linear regression model. The significance exists for all cancer types together and for single analysis for several cancer types like breast, lung, colorectal, prostate, stomach, esophagus, bladder, and many others. The significance is absent for liver cancer and cervix cancer where infectious agents are the main causes. Another research group observed that cancer mortality rate is also higher in accordance with low temperatures [9]. They did not observe any change on the significance level under a multivariate model considering lifestyle factors like diet, alcohol/meat consumption, smoking etc. The same group confirmed that cold is an independent risk factor for several types of cancer, analysing data for different USA populations living in different temperature zones [9]. Two other studies are of special interest. The first is related with prostate cancer where colder environmental temperature has been associated with an overall higher prevalence of prostate cancer [10]. The second one is a USA state-wise analysis where a negative correlation between environmental temperature and thyroid cancer incidence has been reported [11]. This report also suggested that living in the colder state of Alaska gives a two-times risk for thyroid cancer when compared to the warmer state of Texas. Lastly, some interesting cancer data are observed for the cold Nordic countries (Fig. 1). The five Nordic countries are among the first ranked 37 countries (20% of all countries) for incidence of all cancers together and for many individual cancer types [12].

Seki et al. [1], provided data related with colorectal cancer, fibrosarcoma, breast cancer, melanoma, and pancreatic ductal adenocarcinoma. All the experiments seem to be accurate and reliable. The authors state clearly in their abstract: *“We anticipate that cold exposure and activation of brown adipose tissue through any other approach, such as drugs and devices either alone or in combination with other anticancer therapeutics, will provide a general approach for the effective treatment of various cancers”*. Indeed, careful clinical trials could be designed for testing the effect of cold on tumor growth or the effect of anti-cancer drugs under cold conditions. Of course, the immune system must be carefully assessed in cancer patients under cold conditions. Immunity must remain active and not compromised.

There are huge differences when comparing tumor growth between animals living in 4 °C and 30 °C or between animals living in 22 °C and 31 °C. In the first case, activation of brown adipose tissue metabolism can make the difference for tumor growth at 4 °C. In the second case, mild suppression of the immune system at 22 °C is probably the crucial factor for tumor growth. Obviously, immune suppression at very low temperatures is of secondary importance since high adipose tissue metabolism rate can effectively shrink the tumors. Additionally, different mouse strains may display different results. The same can be observed in human populations. Different genetic background or even a different geographic temperature zone can influence the effect that has been showed by Seki et al. [1]. The effect of the cold housing conditions for rodents’ physiology and disease is summarized in some interesting reviews. Different housing temperatures can drive to different experimental results, not only for cancer, but also for other diseases that are being studied in animals [13,14].

The high adaptability of cancer cells must not be underestimated. Tumors may demonstrate a reduced growth under low temperatures, but cancer cells can potentially adapt to the cold environment. They could even become more aggressive. In any case, the effect of temperature on cancer worth further investigation.

## CRediT authorship contribution statement

**Konstantinos Voskarides:** Conceptualization, Writing – original draft.

## Declaration of Competing Interest

The authors declare that they have no known competing financial interests or personal relationships that could have appeared to influence the work reported in this paper.
